# A Langevin equation that governs the irregular stick-slip nano-scale friction

**DOI:** 10.1038/s41598-019-48345-4

**Published:** 2019-08-29

**Authors:** M. Jannesar, A. Sadeghi, E. Meyer, G. R. Jafari

**Affiliations:** 1 Department of Physics, Shahid Beheshti University, G.C., Evin, Tehran, 19839-63113 Iran; 2 School of Nano Science, Institute for Research in Fundamental Sciences (IPM), 19395-5531 Tehran, Iran; 3 Department Physik, University of Basel, Klingelbergstr. 82, 4056 Basel, Switzerland

**Keywords:** Imaging techniques, Statistical physics, Surfaces, interfaces and thin films

## Abstract

Friction force at the nanoscale, as measured from the lateral deflection of the tip of an atomic force microscope, usually shows a regular stick-slip behavior superimposed by a stochastic part (fluctuations). Previous studies showed the overall fluctuations to be correlated and multi-fractal, and thus not describable simply by e.g. a white noise. In the present study, we investigate whether one can extract an equation to describe nano-friction fluctuations directly from experimental data. Analysing the raw data acquired by a silicon tip scanning the NaCl(001) surface (of lattice constant 5.6 Å) at room temperature and in ultra-high vacuum, we found that the fluctuations possess a Markovian behavior for length scales greater than 0.7 Å. Above this characteristic length, the Kramers-Moyal approach applies. However, the fourth-order KM coefficient turns out to be negligible compared to the second order coefficients, such that the KM expansion reduces to the Langevin equation. The drift and diffusion terms of the Langevin equation show linear and quadratic trends with respect to the fluctuations, respectively. The slope 0.61 ± 0.02 of the drift term, being identical to the Hurst exponent, expresses a degree of correlation among the fluctuations. Moreover, the quadratic trend in the diffusion term causes the scaling exponents to become nonlinear, which indicates multifractality in the fluctuations. These findings propose the practical way to correct the prior models that consider the fluctuations as a white noise.

## Introduction

Controlling the friction is an essential part of the emerging technology in nano-scale mechanical devices. The fact of the matter is that in such length scales the fundamentals of friction could be better pronounced^1,2^. Pioneering studies in nano-friction employed the classical model that Tomlinson^3^ and Prandtl^4^ invented originally to describe the dynamics of deformations in atomic crystals. In the PT model, a single nano-asperity that moves across a clean surface, sticks repeatedly to the sites that minimizes its interaction potential energy with the atomic lattice. The elastic force by the spring that drags the asperity detaches it from the sticking site. After a slip, the asperity sticks to the next minimum energy site. Usually, this regular stick-slip behavior of nano-friction has the same periodicity as the lattice constant of the surface^5–8^.

Since the advent of atomic force microscopy (AFM), experiments on nano-friction have shown an irregular quasi-periodic trend which deviates from the basic PT model. The fluctuations may originate from different physical or experimental noises. Thermal effects is one of the main sources of fluctuations at finite temperatures^9–13^. It causes an early jump of the AFM tip to the next minimum energy site by overcoming the finite potential energy barrier. To address thermal effects, researchers commonly use thermally activated PT model which considers the stochastic nature of thermal fluctuations as a Gaussian white noise^14^. This formulation justifies several experimental observations, e.g. the logarithmic dependence of the friction force on the tip speed^14^, or the stochastic nature of the maximum friction force^15^. Other sources such as instrumental noises and lattice deficiency may contribute to nano-friction measurements and should therefore be taken into account^16,17^. In this regard, Dong *et al*.^16,18^ proposed a model which considers instrumental and thermal noises as a white noise. In another series of works, Labuda *et al*.^19–21^ divided the existing noises in a typical AFM experiment into three categories: detection noise, thermal noise, and displacement noise. They determined the stochastic properties of each category by the power spectral method and studied the effect of each individual noise source by simulation. However, the contribution of each category to the nano-friction data was not clarified.

In the present study we aim to shed some light into the nature of the overall fluctuations without concentrating on the origin of the fluctuations or determining the individual contributions. This is in a sense that we find an equation that governs the fluctuations and to some extent provides an insight on the evolution of the fluctuations. In fact, we intend to look inversely into the nano-friction fluctuations: Instead of imposing a model and comparing its prediction with the experimental observations, we directly analyze the data from a typical atomic scale friction experiment to obtain a governing equation for the nano-friction fluctuations. The resultant equation will include the overall effect of all the experimental parameters.

## Results and Discussion

**Figure 1:**
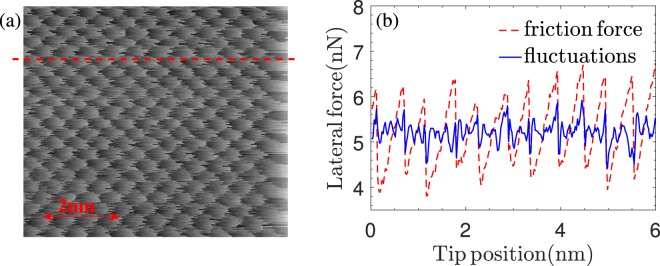
(**a**) Raster image of the friction force data used in this study containing 256 × 256 data points measured by means of a silicon tip scanning the NaCl(001) surface at room temperature along the [100] direction. (**b**) Friction force (raw) and fluctuations (de-trended) versus tip position along the scan line indicated by a dashed line in (**a**).

The raw data are acquired in an earlier experiment using a home-built AFM, where the cleaned (001) surface of NaCl crystal is scanned at room temperature and in the ultra-high vacuum by a pyramidal sharp silicon tip (of radii less than 10 nm) at a scan velocity of 13 nm/s. For technical details of this method with which the scan line can be very precisely aligned with the [100] direction of the NaCl surface refer to ref.^22^. As the test case, we analyze the data set whose correlation and multifractality features have been recently investigated^23^. The set consists of 256 × 256 data points uniformly distributed on a 6 × 6 nm^2^ area, as visualized in Fig. 1(a). In other words, the data is grouped to 256 sets (scan lines) each containing 256 data points with a spatial resolution of 0.23 Å. Along each scan line, being parallel to the [100] crystallographic direction, the tip visits at least ten Na and ten Cl ions, alternatively. Figure 1(b), showing the friction force versus the tip position for the arbitrary scan line indicated by a dashed line, reveals a quasi-periodic (sawtooth-like) trend with the same periodicity as the NaCl lattice constant, i.e. 0.56 nm. To extract fluctuations from the lateral force data, we first eliminate this trend. The corresponding de-trended signal is shown by a solid line line in Fig. 1(b). See Methods for details of the de-trending procedure.

In the following, we determine the Markov length *l*_mar_ of the de-trended data, above which the stochastic process that describes the nano-friction fluctuations will constitute a Markov process. The necessary condition to hold the Markov property is to satisfy Chapman-Kolmogorov equation, namely

1 $$p(f,x|\,{f}_{0},{x}_{0})=\sum _{f^{\prime} }\,p(f,x|\,f^{\prime} ,x^{\prime} )p(f^{\prime} ,x^{\prime} |\,{f}_{0},{x}_{0})$$

where *f* denotes our de-trended nano-friction signal corresponding to the tip position *x*, while ${x}_{0} < x^{\prime}  < x$^24^. In practice, a simple way to estimate the Markov length is to verify the magnitude of the difference between the left and right hand sides of equation (1), namely

2 $$S=|p(f,x|\,{f}_{0},{x}_{0})-\sum _{f^{\prime} }\,p(f,x|\,f^{\prime} ,x^{\prime} )p(f^{\prime} ,x^{\prime} |\,{f}_{0},{x}_{0})|.$$

**Figure 2:**
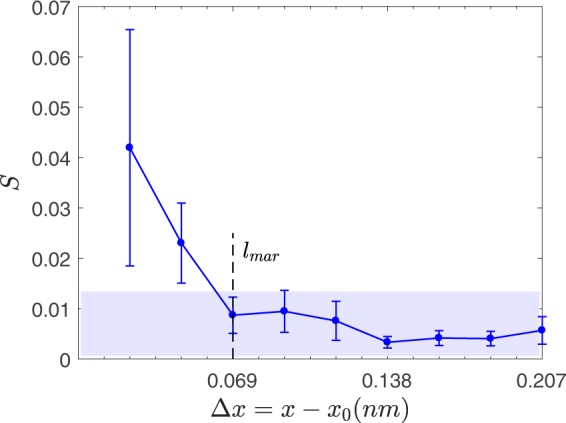
Estimation of *l*_mar_ from the variation of *S*, equation (2), with respect to ${\rm{\Delta }}x=x-{x}_{0}$. The shadow indicates the estimated error bound, from which ${l}_{{\rm{mar}}}=0.07\,{\rm{nm}}$ is obtained.

Then *l*_mar_ is approximately given by the spatial difference Δ$x=x-{x}_{0}$ of de-trended signals *f* and *f*_0_ for which *S* vanishes^25^. Figure 2 shows the variation of *S* with respect to Δ$x=x-{x}_{0}$ for our de-trended nano-frictional signal. Assuming an error bound of 0.014 based on the asymptotic behavior of *S*, one obtains *l*_mar_ = 0.7 Å, meaning that for length scales greater than this value the fluctuations establish a Markov process and conform to Chapman-Kolmogorov equation.

The differential form of equation (1) is known as Kramers-Moyal expansion

3 $$\frac{\partial }{\partial x}p(f,x|\,{f}_{0},{x}_{0})=\mathop{\sum }\limits_{k=1}^{\infty }\,{(-\frac{\partial }{\partial f})}^{k}{D}_{k}(f,x)p(f,x|\,{f}_{0},{x}_{0})$$

where the Kramers-Moyal coefficients *D*_*k*_ are introduced from conditional moments when ${\rm{\Delta }}x\to 0$^26,27^

4 $${D}_{k}=\mathop{\mathrm{lim}}\limits_{{\rm{\Delta }}x\to 0}\frac{1}{K!{\rm{\Delta }}x}\,{\int }_{+\infty }^{-\infty }\,{(f^{\prime} -f)}^{k}p(f^{\prime} ,x+{\rm{\Delta }}x|\,f,x)df^{\prime} .$$

According to Pawula’s theorem, if the forth order coefficient vanishes, then ${D}_{k}\mathrm{=0}$ for all $k\geqslant 3$ and the Kramers-Moyal expansion reduces to Fokker-Planck equation^28,29^. In fact, the Fokker-Planck equation describes the evolution of probability density function of the nano-friction fluctuations that is generated by the Langevin equation

5 $$\frac{\partial f(x)}{\partial x}={D}_{1}(f,x)+\sqrt{{D}_{2}(f,x)}{\rm{\Gamma }}(x),$$

where *D*_1_ and *D*_2_ are called drift and diffusion terms, respectively. The Langevin force $${\rm{\Gamma }}(x)$$ is an uncorrelated white noise with Gaussian distribution satisfying the fluctuation-dissipation theorem $$\langle {\rm{\Gamma }}(x)\rangle =0$$ and $$\langle {\rm{\Gamma }}(x){\rm{\Gamma }}(x^{\prime} )\rangle =$$$$2\delta (x-x^{\prime} )$$.

Multiplying Kramers-Moyal equation by *f*^*  n*^(*x*) and integrating over *f*(*x*) yields the equation that describes the behavior of the moments $\langle \,{f}^{n}(x)\rangle$ (structure function). Assuming a simple case of ${D}_{k}(f,x)={d}_{k}\,{f}^{k}$ (with *d*_*k*_ being a constant), we have^30^

6 $$-\,x\frac{\partial }{\partial x}\langle \,{f}^{n}(x)\rangle =\mathop{\sum }\limits_{k=1}^{n}\,\frac{n!}{(n-k)!}{d}_{k}\langle \,{f}^{n}(x)\rangle .$$

The latter represents the scaling behaviour of the structure function. Considering $\langle \,{f}^{n}(x)\rangle ={x}^{{\xi }_{n}}$ and assuming that only *d*_1_ and *d*_2_ are non-zero, the scaling exponents are obtained as^31,32^

7 $${\xi }_{n}=-\,{d}_{1}n-{d}_{2}n(n-1),$$

which shows the non-linear behavior of the scaling exponents and therefore the multi-fractality of the system. On the other hand, a constant *D*_2_ would yield to a linear ${\xi }_{n}$ with respect to *n*, which demonstrates a mono-fractal behavior. So *d*_1_ is somehow equivalent to the Hurst exponent.

**Figure 3:**
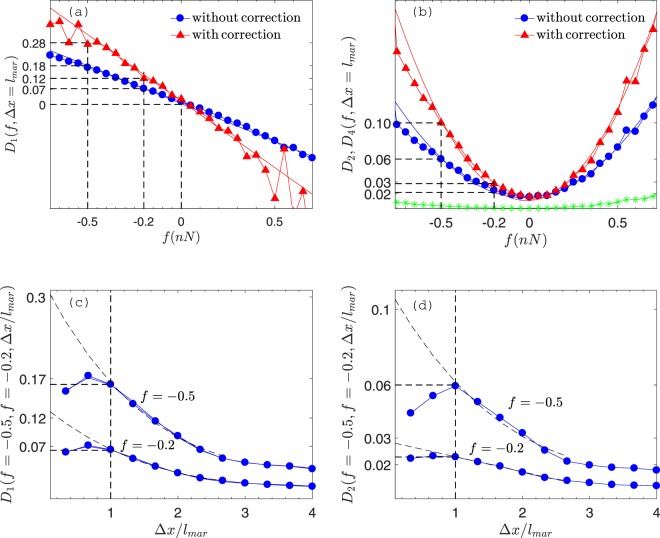
(**a**,**b**) The variation of drift *D*_1_ and diffusion *D*_2_ coefficients with respect to nano-friction fluctuations *f* in two conditions: with (triangles) or without corrected term (dots). For both conditions, drift and diffusion terms show linear and quadratic trends, respectively. In (**b**) the fourth order coefficient *D*_4_ (asterisks) is also shown which is negligible compared with *D*_2_. (**c**,**d**) Step size Δ*x* dependence of *D*_1_ and *D*_2_ for *f* = −0.2 nN and *f* = −0.5 nN.

Figures 3(a,b) display the behaviour of the Kramers-Moyal coefficients ${D}_{k}(f,{\rm{\Delta }}x={l}_{{\rm{mar}}})$ for *k* = 1, 2, and 4. One sees a linear trend for *D*_1_ and a quadratic trend for *D*_2_. The linear dependence of *D*_1_ (dots in (a)) implies that the conditional moment tends to data average (zero) linearly. Also, the quadratic change in *D*_2_ (dots in (b)) is associated with higher variances in data points further from the average. The fourth order coefficient *D*_4_ (asterisks in (b)) is negligible compared with *D*_2_. So the Kramers-Moyal expansion collapses to Fokker-Planck equation.

As expalined in Methods, since the step size Δ*x* is finite in practice while it should be zero to obtain the coeffiecents from equation (4), we apply two different approaches and compare their results in the following.

First, the second-order expansion for moments given by^33^

8 $$\begin{array}{rcl}\langle \,f-{f}_{0}|\,{f}_{0}\rangle  & = & {\rm{\Delta }}x{D}_{1}+\frac{1}{2}{\rm{\Delta }}{x}^{2}[{D}_{1}({D}_{1})^{\prime} +{D}_{2}({D}_{1})^{\prime\prime} ]+{\mathscr{O}}({\rm{\Delta }}{x}^{3}),\\ \langle {(f-{f}_{0})}^{2}|\,{f}_{0}\rangle  & = & 2{\rm{\Delta }}x{D}_{2}+{\rm{\Delta }}{x}^{2}[{({D}_{1})}^{2}+2{D}_{2}({D}_{1})^{\prime} \\  &  & +\,{D}_{1}({D}_{2})^{\prime} +{D}_{2}({D^{\prime\prime} }_{2})]+{\mathscr{O}}({\rm{\Delta }}{x}^{3})\end{array}$$

is evaluated. The triangle points in Figs. 3(a,b) show the corrected *D*_1_ and *D*_2_ coefficients in ${\rm{\Delta }}x={l}_{{\rm{mar}}}$. The two dashed lines correspond to two de-trended nano-friction values *f* = −0.2 and −0.5 nN. The value of corrected terms are 0.12 and 0.28 for *D*_1_ and 0.03 and 0.10 for *D*_2_, respectively.

Second, to use extrapolation, we plot *D*_1_ and *D*_2_ with respect to step size Δ*x* for *f* = −0.2 and −0.5 nN (dot points in Figs. 3(c,d)). We observe that both coefficients decrease with Δ*x* for ${\rm{\Delta }}x\geqslant {l}_{{\rm{mar}}}$. This is reasonable, because for ${\rm{\Delta }}x\geqslant {l}_{{\rm{mar}}}$ and in stationary condition, $p(f^{\prime} ,x+{\rm{\Delta }}x|\,f,x)$ reduces to $p(f^{\prime} ,x)$ and the integral in equation (4) becomes independent of Δ*x*. However, for ${\rm{\Delta }}x\leqslant {l}_{{\rm{mar}}}$, the behavior obviously changes because, for length scales smaller than Markov length, equation (4) becomes invalid. So we extrapolate *D*_1_ and *D*_2_ for ${\rm{\Delta }}x\to 0$. For *f* = −0.2 nN, the estimated values obtained for *D*_1_ and *D*_2_ are 0.12 and 0.03 respectively. These values are 0.3 and 0.10 for *f* = −0.5 nN.

The values obtained from both approaches closely match. So we obtain a good approximation for drift and diffusion terms. Based on Figs. 3(a,b), we report the drift and diffusion equations *D*_1_ = (−0.61 ± 0.02) *f* and *D*_2_ = (0.37 ± 0.01) *f* ^2^ respectively (the errors are those of the least squares fitting procedure, while the thin lines show the fitted curves). Finally, we form the Langevin equation for nano-friction fluctuations

9 $$\frac{\partial f(x)}{\partial x}=-\,0.61f+\sqrt{0.37{f}^{2}}{\rm{\Gamma }}(x).$$

**Figure 4:**
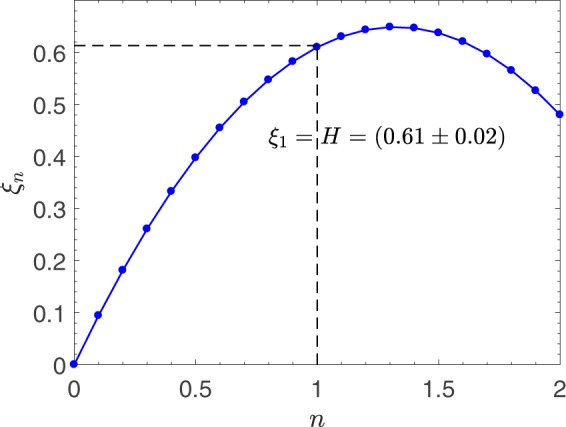
The non-linear behavior of scaling exponents *ξ*_*n*_ of nano-friction experimental data with respect to *n*.

The drift and diffusion terms of the governing Langevin equation directly relates to scaling exponents. The linear drift contains correlation information. The slope of this term, 0.61 ± 0.02, is equivalent to system’s Hurst exponent and represents the degree of correlation in the fluctuations. The diffusion term, which corresponds to the stochastic part of Langevin equation, contains the information of the combined noise contribution of the nano-friction data (thermal noise, instrumental noise, etc.). The quadratic behavior of diffusion term indicates the nonlinearity of scaling exponents in the nano-friction fluctuations. By substituting the drift and diffusion coefficients into equation (7), the scaling exponents of the fluctuations is obtained as ${\xi }_{n}=0.61n-0.37n(n-1)$. The nonlinear behavior of scaling exponents ${\xi }_{n}$ with respect to moments *n* is associated with the multi-fractality of nano-friction data (Fig. 4). This means that the scaling exponents depend on the order of the moments and thus the Hurst exponent alone is not enough to describe the scaling behavior of the fluctuations. The origin of the observed multi-fractality can be due to non-Gaussian behavior of the nano-friction fluctuations.

Our model is primarily thought for the case of atomic friction, where a regular atomic lattice is imaged and stick-slip pattern can be recognized. Experimental conditions and parameters such as sample material, tip material, tip size and velocity, temperature and so on greatly affect the nano-friction data. Since we directly obtain the governing equation from empirical data, all of the contributing parameters are included. A different set of data yields to a different drift and diffusion terms in the resultant Langevin equation. For example, we applied our method to the friction force data acquired on the surface of highly oriented pyrolytic graphite (HOPG) at room temperature and in UHV with a scan velocity of 60 nm/s^15^. We found that the fluctuations in this case establish a Markov process for length scales greater than 0.03 nm, and satisfy the Langevin equation $\frac{\partial f(x)}{\partial x}=-\,0.38f+\sqrt{0.25{f}^{2}}{\rm{\Gamma }}(x)$. As listed in Table 1, different experimental conditions (sample surface, scan velocity and normal load) yield different coefficients for the drift and diffusion terms of the Langevin equation.

**Table 1:** Drift and diffusion terms in Eq. (5) for two typical experimental conditions.

Surface	Scan velocity	Normal load	Drift	Diffusion
NaCl(001)	13 nm/s	3 nN	(−0.61 ± 0.02) *f*	(0.37 ± 0.01) *f*^2^
HOPG(0001)	60 nm/s	11 nN	(−0.38 ± 0.02) *f*	(0.25 ± 0.01) *f*^2^

## Conclusions

By directly studying the irregular data of the stick-slip behavior of dragging a tip on the clean NaCl(001) surface as a stochastic data set, the de-trended nano-friction signal is modeled in terms of its drift and diffusion contributions. We revealed that the fluctuations comply with a Langevin equation while the scaling exponents corresponding to the drift and diffusion terms are determined by equation (7). Generally, the drift term is connected to the Hurst exponent while the diffusion coefficient is a measure of the multifractality and non-Gaussian distribution of the fluctuations. The observed multifractality in the fluctuations confirms the suggestion of the previous works^20,23,34^ that the combined noise contribution in nano-friction data cannot be considered as a simple random white noise. The significance of introducing this general framework for analyzing experimental nano-friction is that we show that how one can apply the Kramers-Moyal approach to obtain the governing Langevin equation for any nano-friction fluctuation data, without knowing the contribution and effect of each noise source separately. The variation of experimental conditions or effective parameters causes variations in the drift and/or diffusion terms of the Langevin equation which can be immediately captured in our proposed framework. Focusing on the behaviour of the irregularities inherent in nano-frictional signals regardless of their physical or instrumental sources, we considered the collective effects of all such parameters in this study. However, one can selectively change only one of such parameters in experiment and identifies its direct effect on the correlation and multifractality of the signal. For example, the physical interpretation of the drift term and its dependence on individual experimental parameters can in principle be explored if the parameter in question is altered while keeping other ones fixed. The periodic trend of the original friction signal is known to be related to the atomic structure of the scanned crystal surface. Now, the methodology introduced here opens the door to investigate the effect of the physical properties of the scanned surface or the probe on the multifractality nature of the friction force. Moreover, environment parameters and measurement concerns like temperature or electronic instruments, may have an effect on the stochastic properties of the friction signal, the effect of which can be determined in the same way.

## Methods

The quasi-periodic trend in the acquired nano-friction signal must be eliminated prior to analysing its fluctuations. The technical details of both steps are given in the follwing.

### De-trending

A number of de-trending methods are available^35,36^. Fourier transformation^37,38^ as a natural way for removing sinusoidal trends, cannot completely eliminate the quasi-periodic stick-slip trend in the nano-friction data. The singular value decomposition (SVD) technique suits better the present application. It eliminates not only periodic but also quasi-periodic trends from the series which are characterized by more than a single peak in the power spectrum^39,40^. In the present case two distinguishably dominant frequencies (*p* = 2 peaks) are present in the power spectrum for every scan line. Applying SVD for removing the quasi-periodic trends from the signal is based on the fact that the trends are described by the eigenvectors of the interaction matrix corresponding to its largest eigenvalues. To filter out the trends from the raw friction forces we first construct the matrix ${({\gamma }_{1},{\gamma }_{2},\ldots ,{\gamma }_{d})}^{T}$, where *d* is called the embedding dimension, from the *N* = 256 data points $\{{f}_{1},{f}_{2},\ldots ,{f}_{256}\}$ for each scan line. Since the dimension of the matrix should be much greater than the number of peaks, we set *d* = 100. The rows of the matrix are vectors ${\gamma }_{k}=({F}_{k},{F}_{k+1},\ldots ,{F}_{k+N-(d-1)})$. The filtered matrix is gained simply by setting the 2*p* + 1 largest singular values of the original matrix equal to zero. The rows of the new matrix give the de-trended data series, as the one shown by a solid line in Fig. 1(b). We finally shift the average of each de-trended data set to zero and work with the remained signal (fluctuations) series $\{{f}_{1},{f}_{2},\ldots ,{f}_{256}\}$ for each scan line.

### Fluctuation analysis

From the de-trended data series, we calculate the Kramers-Moyal coefficients *D*_*k*_, defined by equation (4), to characterize the fluctuations nature. It should be noted that not all the coefficients need to be evaluated in practice. The Pawula’s theorem simplifies the procedure: the Kramers-Moyal expansion stops after the second term if the fourth order coefficient vanishes. In other words, it turns out that *D*_4_ is vanishingly small in the present case and thus evaluating only *D*_1_ and *D*_2_ would suffice. We estimate the coefficients *D*_1_, *D*_2_ and *D*_4_ by calculating the conditional moment at ${\rm{\Delta }}x={l}_{{\rm{mar}}}$ for every row of the de-trended nano-friction raster image and then averaging over all the rows. According to equation (4) an accurately determination of *D*_*k*_ is gained at the limit ${\rm{\Delta }}x\to 0$ while in practice the step size Δ*x* is finite. To compensate for this limitation, we apply two different approaches and compare their results. First, we use the second-order expansion for moments to obtain more accurate results in ${\rm{\Delta }}x={l}_{{\rm{mar}}}$. Second, we plot *D*_*k*_ with respect to Δ*x* and then approximate the desired quantity by extrapolating the plot to Δ*x* = 0. A good agreement between the results from the two approaches is a signature of the reliable numerical results.
